# Diagnostic accuracy differences in detecting wound maceration between humans and artificial intelligence: the role of human expertise revisited

**DOI:** 10.1093/jamia/ocaf116

**Published:** 2025-07-16

**Authors:** Florian Kücking, Ursula H Hübner, Dorothee Busch

**Affiliations:** Research Center of Health and Social Informatics, Osnabrück University of Applied Sciences, 49009 Osnabrück, Germany; Research Center of Health and Social Informatics, Osnabrück University of Applied Sciences, 49009 Osnabrück, Germany; Research Center of Health and Social Informatics, Osnabrück University of Applied Sciences, 49009 Osnabrück, Germany; Department of Dermatology, University Hospital, Friedrich-Alexander University Erlangen-Nürnberg, 91012 Erlangen, Germany

**Keywords:** artificial intelligence, human, wound healing, wound images, diagnostic accuracy, interrater-reliability

## Abstract

**Objective:**

This study aims to compare the diagnostic abilities of humans in wound image assessment with those of an AI-based model, examine how “expertise” affects clinicians’ diagnostic performance, and investigate the heterogeneity in clinical judgments.

**Materials and Methods:**

A total of 481 healthcare professionals completed a diagnostic task involving 30 chronic wound images with and without maceration. A convolutional neural network (CNN) classification model performed the same task. To predict human accuracy, participants’ “expertise,” ie, pertinent formal qualification, work experience, self-confidence, and wound focus, was analyzed in a regression analysis. Human interrater reliability was calculated.

**Results:**

Human participants achieved an average accuracy of 79.3% and a maximum accuracy of 85% in the formally qualified group. Achieving 90% accuracy, the CNN performed better but not significantly. Pertinent formal qualification (β  =  0.083, *P* < .001) and diagnostic self-confidence (β  =  0.015, *P* = .002) significantly predicted human accuracy, while work experience and focus on wound care had no effect (R^2^ = 24.3%). Overall interrater reliability was “fair” (Kappa = 0.391).

**Discussion:**

Among the “expertise”-related factors, only the qualification and self-confidence variables influenced diagnostic accuracy. These findings challenge previous assumptions about work experience or job titles defining “expertise” and influencing human diagnostic performance.

**Conclusion:**

This study offers guidance to future studies when comparing human expert and AI task performance. However, to explain human diagnostic accuracy, “expertise” may only serve as one correlate, while additional factors need further research.

## Introduction

The development of artificial intelligence (AI)-powered clinical decision support systems (CDSS) in highly visual medical fields has experienced significant growth in recent years^1–4^—for analyzing images or videos,^5^ supporting clinical diagnosis,^6^ and detecting diseases.^7^^,^^8^ With the improvement of deep learning approaches and the use of larger datasets, algorithms are now able to achieve the same results as experts in certain medical tasks.^9^ In dermatology, where visual judgment is key, AI systems have become increasingly reliable in classifying dermatological findings, eg, various types of skin cancer as well as inflammatory diseases and chronic wounds.^10–16^ Studies that have been conducted on wounds have shown that AI models are able to detect visual signs of potential healing complications, such as maceration (the swelling and softening of tissue due to prolonged contact with fluid^17^) or infection.^15^^,^^18–20^ Therefore, AI opens up new possibilities for wound assessments where, unlike skin cancer, the findings cannot be or are usually not histologically verified.

While the advances of AI enabled diagnostics are undeniable, the question remains as to whether and when they are performing better than humans in certain tasks. A systematic review and meta-analysis by Liu et al., including 82 studies, compared the diagnostic accuracy of medical image-based disease classification by deep learning algorithms and healthcare professionals. They found their performance to be comparable (sensitivity of 87% for AI vs 86.4% for humans).^21^ The systematic review by Shen et al.—based on 9 articles—concluded that AI models performed comparably to medical experts and outperformed less experienced clinicians.^22^ The validity of these comparisons, however, suffered from the fact that human performance was only investigated in very small groups of clinicians, ranging from 2 to 42. Only the study by Brinker et al. established a benchmark for human melanoma detection in dermoscopic images including 157 dermatologists.^23^ In a subsequent study, they also compared the diagnostic results of a convolutional neural network (CNN) with those of 145 dermatologists, showing that the CNN performed on par with the average dermatologist. Still, 19 out of 145 human experts achieved a higher sensitivity than the CNN,^24^ thereby indicating a differential effect.

Besides the low number of clinicians in most studies, only limited information about the “human experts” could be found, although “experience” has emerged as a candidate to explain differential effects. In one of the larger studies, the CNN significantly outperformed physicians with less than 10 years of experience, but revealed no significant difference compared to the most experienced doctors.^25^ The lack of information about the human experts could be illustrated very well in the systematic review by Shen et al., which revealed that, in 7 out of 9 studies, “human expertise”—as one of the most outstanding characteristics—was coded by job title at most, and not at all in the rest of the cases.^22^ In other individual studies, clinical experience was expressed as the number of years working in the field.^25^^,^^26^ While all of these findings originate from dermatological studies in skin cancer, studies of other skin conditions, such as wounds, render a similar picture.^27^

Expressing human diagnostic ability as the mean of expert opinions overlooks the group’s diversity and assuming job title to represent expertise may simplify matters. Experts can greatly differ in education, training, years of experience, work focus, and diagnostic self-confidence as well as demographic factors such as age, gender, and healthcare setting. To our knowledge, these characteristics have not yet been analyzed in the context of comparing human and AI-based diagnostic performance. Knowledge about group differences would yield more nuanced insights and would help identify those individuals who can benefit the most from the assistance of an AI-based system.

Therefore, the aim of the present study is to compare human diagnostic ability in wound image assessment with that of an AI-based model. Hereby, we also seek to find the characteristics that are associated with human diagnostic judgments. Furthermore, we are interested in the overall interrater reliability, and particularly between different groups, to better understand the homogeneity or heterogeneity regarding their judgment.

## Materials and methods

### Study design and participants

To address the research questions, we conducted a clinical study requiring clinicians and an AI system to determine the presence or absence of maceration in images of chronic wounds. Our aim was to operationalize human expertise by the set of the following variables: *pertinent formal qualification*, *work experience*, and *diagnostic self-confidence* in detecting maceration. The variable as to whether there had ever been a *focus on wound care* in their professional life (eg, working as a specialist in a wound care center or with a home care provider focused on wound care) rounded out this set. The selection of these variables was informed by the Theory of Expert Competence,^28^^,^^29^ which involves among others domain knowledge and psychological expert traits, including self-confidence. To choose the variables, other studies that point at extensive deliberate practice^30^ and formal qualification^31^ were also drawn upon. In wound care, there is a wealth of studies, recommendations, and guidelines for assessing and treating wounds.^32^^,^^33^ This knowledge underpins the understanding of *pertinent formal qualification* in wound care.

In addition, clinicians were also characterized by *age*, *gender*, and *healthcare setting* (outpatient or inpatient). The complete questionnaire is shown in Appendix S1.

Each clinician received 30 wound images via an electronic form (LimeSurvey version 6.6.6) capturing the diagnostic decisions and participant characteristics. To avoid sequence effects, the images were displayed in random order. There were no time constraints to complete the task.

The same diagnostic task with the same 30 wound images was presented to a CNN image classification system that had been developed based on a pre-trained MobileNetV2 architecture in a previous study. This model, which was trained on 458 images, demonstrated superior performance in classifying wound images with and without maceration compared to other models. It was selected due to its high efficiency, achieving the highest accuracy (77%) despite having significantly fewer parameters (3.5 million) than more complex architectures. Transfer learning with ImageNet weights, data augmentation, dropout, and early stopping had been applied to reduce overfitting and enhance the generalizability.^15^ The images that the model was trained on were sourced from the same hospital as the images for the maceration detection task, but the 2 datasets were completely distinct.

The participants were recruited from among the physicians, nurses, and other healthcare professionals across Germany between May 2024 and July 2024 through online advertisements in 1893 German hospitals, at a wound congress 2024 with 4628 attendees,^34^ via the professional association “Initiative Chronic Wounds e. V.” (ICW), and the Department of Dermatology at Erlangen University Hospital. None of the clinicians who determined the ground truth took part in the study. The inclusion of participants without pertinent formal qualification in wound care was essential to adequately and realistically reflect the situation in providing wound care. Not uncommonly, patients with chronic skin problems contact this group of health professionals first before they are referred to a specialist.

The study protocol was approved by the Ethics Committee of Osnabrück University of Applied Sciences (no. HSOS/2024/1/1).

### Characteristics and preselection of the wound images

To establish the ground truth for the maceration classification, an ICW-certified wound expert initially selected 110 leg ulcer images with various diagnoses from the University Hospital Erlangen’s EHR, all with a clearly recognizable maceration status. Five additional wound experts (ICW-qualified nursing and medical staff) independently assessed these images. From their unanimous decisions, 15 images with and 15 without maceration were chosen, yielding 30 images in total. All of the images were reviewed by the hospital’s Data Integration Center for anonymity and by a certified wound expert for high image quality.

### Data preparation

All the characteristics of the participants were treated as binary variables to compare the performance metrics and the interrater reliability between different groups. Professional background was classified either as with *pertinent formal qualification* (dermatology residents/specialists or ICW-certified nurses) or without (other physicians/nurses without specialized wound care training). *Work experience* was categorized into participants with up to 5 years and those with more than 5 years of experience.^26^  *Age* was binarized by median split and *diagnostic self-confidence*, assessed using a 10-point scale, and binarized into *low* and *high*. For the regression analysis, *work experience*, *age*, and *diagnostic self-confidence* were treated as continuous variables. Participants with non-patient-care qualifications or incomplete responses were excluded.

### Statistical analysis

Diagnostic performance was evaluated by accuracy, sensitivity (recall), and specificity (mean and 95% confidence interval^35^). Accuracy served as the primary outcome and was tested for significance in multiple paired t-tests. Alpha (0.05) was, therefore, corrected according to the Holm-Bonferroni method. Positive and negative predictive values can be found in Appendix S2.

A multiple linear regression analysis was performed to identify the correlates that might influence the participants’ accuracy in decision making when detecting maceration. Demographic characteristics (*age*, *gender*, *healthcare setting*) as well as objective and subjective professional expertise (*pertinent formal qualification*, *years of work experience*, *focus on wound care*, and *diagnostic self-confidence*) were used as independent variables. All of the indicators met the requirements of multiple linear regression analysis. Appendix S3 contains the diagnostic tests and their results. The results of the statistical analyses were tested at a significance level of *P* < .05.

To evaluate the interrater reliability between the different participant groups in the assessment of maceration, Fleiss’ kappa was calculated for the entire sample and separately by groups.

The statistical analyses were conducted using R (version 4.4.1) with additional packages, including lmtest (version 0.0-40), irr (version 0.84.1), and car (version 3.1-3), to perform advanced modeling, reliability assessments, and diagnostic testing. Diagrams were created using the ggplot2 package (version 3.5.1).

## Results

### Sample characteristics

A total sample of 481 participants was included in the study (Table  1). The sample is balanced with the exception of work experience (more persons with long experience), gender (more females), and healthcare setting (more persons from inpatient care). Bivariate descriptive statistics (phi-coefficient) are shown in Appendix S4. Variables that moderately correlate with each other are age and work experience (phi = 0.35), pertinent formal qualification with diagnostic self-confidence (phi = 0.40) and a focus on wound care (phi = 0.30), and diagnostic self-confidence with a focus on wound care (phi = 0.40).

**Table 1.** Sample characteristics.

Characteristic	N	%	Mean (SD)	Median (IQR)
**Work experience**	481		18.89 (11.46)	18 (18)
Short (≤5 years)	68	14.14		
Long (>5 years)	413	85.86		
**Pertinent formal qualification**				
No	232	48.23		
Yes	249	51.77		
**Focus on wound care**				
No	215	44.7		
Yes	266	55.3		
**Diagnostic self-confidence**	481		6.75 (2.3)	7 (3)
Low	264	54.89		
High	217	45.11		
**Age**	481		41.41 (11.07)	40 (18)
Young (≤40 years)	244	50.73		
Old (>40 years)	237	49.27		
**Gender**				
Male	100	20.79		
Female	381	79.21		
**Healthcare sector**				
Outpatient	45	9.36		
Inpatient	436	90.64		

SD, standard deviation; IQR, interquartile range.

### Human and AI-performance metrics

Overall, human participants achieved an accuracy of 79.3%, a sensitivity of 76.4%, and a specificity of 83%. In comparison, the AI classification model demonstrated an accuracy of 90%, a sensitivity of 93.3%, and a specificity of 86.7%, thereby outperforming the human results on a descriptive level. However, when tested for significance, the accuracy values of humans were only significantly lower than those of the CNN in the groups without *pertinent formal qualification*, low *diagnostic self-confidence*, no *focus on wound care*, and short *work experience* (Table 2).

**Table 2.** Selected metrics of human and AI diagnostic decisions.

Group	N	Sensitivity		Specificity		Accuracy		
		Result	95% CI	Result	95% CI	Result	95% CI	*P* *
**CNN**		0.933	0.68-1.00	0.876	0.60-0.98	0.900	0.73-0.98	
**Overall human experts**	481	0.764	0.75-0.77	0.830	0.82-0.84	0.793	0.79-0.80	.022
**Work experience**								
Short (≤ 5 years)	68	0.698	0.67-0.72	0.777	0.75-0.80	0.731	0.71-0.75	**.002**
Long (> 5 years)	413	0.776	0.77-0.79	0.838	0.83-0.85	0.804	0.80-0.81	.035
**Pertinent formal qualification**								
No	232	0.705	0.69-0.72	0.769	0.75-0.78	0.732	0.72-0.74	**<.001**
Yes	249	0.822	0.81-0.83	0.883	0.87-0.89	0.850	0.84-0.86	.275
**Focus on wound care**								
No	215	0.730	0.72-0.74	0.794	0.78-0.81	0.758	0.75-0.77	**.003**
Yes	266	0.792	0.78-0.80	0.858	0.85-0.87	0.822	0.81-0.83	.089
**Diagnostic self-confidence**								
Low	264	0.726	0.71-0.74	0.789	0.78-0.80	0.754	0.74-0.76	**.003**
High	217	0.812	0.80-0.82	0.878	0.87-0.89	0.842	0.83-0.85	.198
**Age**								
Young (≤ 40 years)	244	0.754	0.74-0.77	0.820	0.81-0.83	0.783	0.77-0.79	.015
Old (> 40 years)	237	0.775	0.76-0.79	0.840	0.83-0.85	0.804	0.79-0.81	.033
**Gender**								
Male	100	0.772	0.75-0.79	0.850	0.83-0.87	0.806	0.79-0.82	.045
Female	381	0.762	0.75-0.77	0.824	0.81-0.83	0.790	0.78-0.80	.018
**Healthcare sector**								
Outpatient	45	0.803	0.77-0.83	0.867	0.84-0.89	0.832	0.81-0.85	.153
Inpatient	436	0.760	0.75-0.77	0.826	0.82-0.84	0.789	0.78-0.80	.018

* *P*-values are derived from paired t-tests comparing each group to the CNN. The significance threshold was adjusted for multiple comparisons using the Bonferroni correction (α = 0.0034). Significant values are shown in bold.

**Figure 1.**
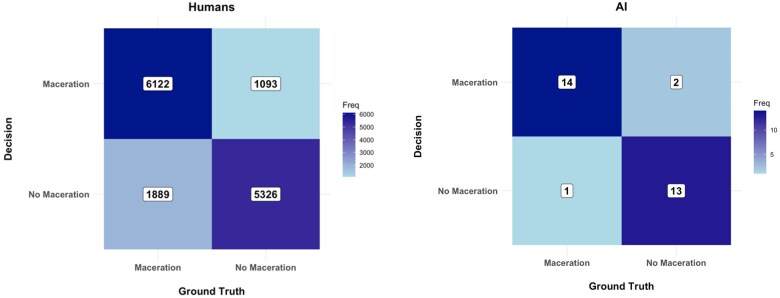
Confusion matrix for human and AI model decisions.

Figure 1 shows the confusion matrix for the clinicians and the AI model.

Participants with a *pertinent formal qualification* demonstrated the highest values in all metrics with a higher accuracy (85% vs 73.2%), sensitivity (82.2% vs 70.5%), and specificity (88.3% vs 76.9%) compared to those without such a qualification (Table 2).

A similar pattern emerged for participants with high *self-confidence in diagnosing maceration* compared to those who lacked this confidence (Table 2). Furthermore, the analysis revealed that the subgroup (n = 160) with the *wound care qualification plus high self-confidence* exhibited a sensitivity of 83.3%, specificity of 89.7%, and accuracy of 86.3%, thereby reaching the highest values in the groups of humans on a descriptive level. The negative predictive value (NPV) and positive predictive value (PPV) results for the CNN and all groups can be found in Appendix S2.

### Influencing factors on human accuracy—regression analysis

To better understand which group differences were significant, a multiple linear regression analysis on the overall diagnostic accuracy was conducted. The findings (Table 3) show that *pertinent formal qualification* (coefficient of 0.083; *P* < .000) and *diagnostic self-confidence* (coefficient of 0.015; *P* = <.001) were significant, while other factors, such as group differences in *work experience* and a *focus on wound care*, were not. The model accounted for 24.3% of the overall variance (R^2^ = 0.243, *P* < .001).

**Table 3.** Influencing factors on the overall correct answers: multiple linear regression results.

Coefficient	b	SEM	T	*P*	95% CI	
					Lower	Upper
**Constant**	0.659	0.042	15.706	**<.001**	0.577	0.741
**Work experience**	0.001	0.001	0.600	.549	−0.001	0.003
**Pertinent formal qualification**	0.083	0.013	6.576	**<.001**	0.057	0.109
**Focus on wound care**	0.002	0.013	0.157	.875	−0.024	0.028
**Diagnostic self-confidence**	0.015	0.003	5.178	**<.001**	0.009	0.021
**Age**	0.000	0.001	−0.161	.872	−0.002	0.002
**Gender**	−0.009	0.014	−0.620	.535	−0.036	0.018
**Healthcare sector**	−0.011	0.019	−0.590	.555	−0.048	0.026
**R^2^**	0.243					
***P***	**<.001**					

Significant values are shown in bold.

### Inter-rater reliability

While the performance metrics indicate the level of correct diagnostic decisions, inter-rater reliability reflects the homogeneity or heterogeneity of groups regarding the decision (Table 4). Fleiss’ Kappa yielded values for the entire sample of 0.391 (*P* < .001), indicating a “fair” agreement according to Landis and Koch.^36^

**Table 4.** Results of the inter-rater reliability Fleiss’ kappa for more than 2 raters based on 30 votes per rater.

Group		Kappa	Raters
**Overall**		0.391	481
**Work experience**	Short (≤5 years)	0.296	68
	Long (>5 years)	0.411	413
**Pertinent formal qualification**	No	0.277	232
	Yes	0.531	249
**Focus on wound care**	No	0.320	215
	Yes	0.457	266
**Diagnostic self-confidence**	Low	0.310	264
	High	0.508	217
**Age**	Young (≤40 years)	0.375	244
	Old (>40 years)	0.411	237
**Gender**	Male	0.422	100
	Female	0.383	381
**Healthcare sector**	Outpatient	0.492	45
	Inpatient	0.383	436

Health professionals with a *pertinent formal qualification* achieved the highest agreement (kappa = 0.531; “moderate”) compared to those without such qualifications (kappa = 0.277; “fair”) followed by persons who were *self-confident* of their diagnostic ability (kappa = 0.508; “moderate”) versus those who were not (kappa = 0.310; “fair”). Other groups that achieved a “moderate” agreement comprised persons with long *work experience* with a *focus on wound care*, working in an *outpatient setting*, and *older* and *male* persons.

## Discussion

### Summary

The present study is the first to compare detailed human characteristics with AI-based diagnostic performance in detecting maceration in chronic wound images. To this end, a large and heterogeneous sample of healthcare professionals was recruited, outnumbering the previous sample sizes.^21^ Based on the selected images, the clinical task was designed to be straightforward and without ambiguities. Although the diagnostic accuracy of the AI model was rather high (90%) and outperformed the humans (79.3%), this difference was not statistically meaningful. In contrast, the poor performing groups, ie, without *pertinent formal qualification*, low *diagnostic self-confidence*, no *focus on wound care*, and short *work experience*, revealed a significantly lower accuracy than the CNN. The best performing groups were those with a *pertinent formal qualification* and high *diagnostic self-confidence*, whereby the ones that combined these 2 characteristics achieved the highest accuracy values among humans on a descriptive level. Significant differences were found in the diagnostic accuracy between the groups with and without a *pertinent formal qualification* and those with a high and low *diagnostic self-confidence* in assessing maceration. *Work experience* and *focus on wound care* had no significant influence. Overall, the inter-rater reliability was found to be “fair,” whereby groups of individuals with a *pertinent formal qualification* and high *diagnostic self-confidence* also reached a higher agreement among themselves than any of the other groups. However, no group agreed at a “substantial” or higher level.

These results challenge the notion that visual wound assessment is a simple task for clinicians, highlighting the impact of individual knowledge and skill. They also open room for AI assisted wound diagnostic tools to augment the diagnostic capabilities of clinicians demonstrating a lower diagnostic performance.

### Human versus AI decisions

To our knowledge, there are no similar studies in wound care for serving as a comparison with our results. Studies in melanoma detection show either the superior or on par performance of an AI model compared to humans.^23^^,^^24^^,^^37^^,^^38^ For melanoma, Brinker et al. found a sensitivity value of 67.2% and specificity of 62.2% for dermatologists in lesion detection compared to the CNN achieving 82.3% sensitivity and 77.9% specificity.^39^ Esteva et al., when evaluating a CNN trained to differentiate malignant melanomas from benign nevi and keratinocyte carcinomas from seborrheic keratoses, found an accuracy of the CNN of 72.1% compared to 2 dermatologists reaching an accuracy of 65.6% and 66%.^16^ Judging by the absolute height of these values, they are lower than the ones we found, which could be explained by the relatively straightforward task in our study.

The CNN in the present study (accuracy of 90%) exhibited a better performance than in the original study, which had reported an accuracy of 77%.^15^ This improvement is likely from the original study’s use of a more challenging dataset for CNN training and validation, featuring images with varying degrees of maceration clarity.^15^

### Influence on human decision making and the nature of “expertise”

There was considerable variability in diagnostic accuracy, ranging from 73.1% (short *work experience*) to 85% (with *pertinent formal qualification*). However, only *formal qualification* and *diagnostic self-confidence* significantly affected human accuracy, thereby suggesting that specialized training and trust in one’s abilities make the difference.

Similar to accuracy, these 2 groups exhibited the largest interrater agreement, again demonstrating their decisive influence. In contrast to intuitive assumptions, neither *work experience* nor *work focus* in wound care were significant factors influencing the quality of the clinical judgment nor showed the highest interrater agreements.

This raises the question of what defines “expertise.” Earlier studies used job title or years of work experience,^21–24^^,^^26^ but our findings indicate that *formal qualifications* plus *self-confidence* are key, while years in healthcare or a wound care focus play secondary roles.

Potentially confounding factors such as *age*, *gender*, and *healthcare setting* appear to have an equally low effect. The significant characteristics of expertise are expressed not only in the accuracy of the diagnostic judgment but also at a descriptive level in the interrater reliability as well as in other quality metrics of human judgment such as sensitivity and specificity.

These results help gain a more thorough understanding of human expertise. They partly corroborate the general literature on human expertise pertaining to formal qualification and domain knowledge,^28^^,^^29^^,^^31^ ie, having a degree in this field accompanied with practical training in real-world settings. The study also supports the psychological trait of being self-confident about one’s own abilities.^29^^,^^40^ Regarding the role of years of experience, our findings support studies with a critical view^28^^,^^41^ rather than those that emphasize their influence.^30^

The percentage of total variance in the regression model is a clear hint at other latent factors not included in the study that can exert influence on the diagnostic performance. Such additional variables could comprise, among others, cognitive skills and decision-making strategies^29^ as well as the risk perception and emotional impact of “regrettable decisions.”^42^ Regret theory might explain why sensitivity is higher than specificity in our study and most of the literature, as overlooking something worthy of treatment could be more regrettable. On top of this, there is a wealth of other known factors from the literature influencing human decision making in healthcare.^43^ Not all of them are of practical interest in the context of the present study as they do not characterize human decision makers. However, some might be relevant and enhance the variance explained, such as the time needed to do the task or time constraints (stress) and their interaction with the level of expertise.^44^

### AI and human decision making

While the significant superiority of AI in this task compared to the low performing humans was demonstrated by the findings, the underlying reasons can only be speculated. The superior performance could be attributed to the fact that it relies on a single training source and, therefore, a single ground truth—albeit heterogeneous regarding the images showing wounds of different diagnoses. In contrast, human experts are subject to their own individual experiences that are grounded on education and the exposition to relevant cases, which is mirrored by the variability of human judgments. A deeper understanding of these performance differences is essential for curating training datasets and designing AI systems that specifically compensate for the human weaknesses observed in everyday clinical practice. Despite this variability, the diagnostic decisions converged, and interrater reliability increased with a high level of relevant education and training plus a good amount of self-confidence.

In AI-assisted diagnostics, users without the necessary qualification, low self-confidence, no focus on wound care, and short work experience may benefit most from AI support. However, they are the groups that are more susceptible to accepting false machine recommendations, which is a circumstance described as automation bias.^45^^,^^46^ This emphasizes the importance of reliable AI systems and high-quality ground truth, especially in areas like wound diagnostics, where ground truth labels heavily rely on clinical expertise. Variations in annotator expertise can have a significant impact on model performance. For example, Ramachandram et al. showed that, even among trained professionals, inconsistencies in wound labeling led to noticeable declines in segmentation accuracy. Their results clearly emphasize the need for expert-generated and consistent annotations when developing clinically robust AI systems.^47^

Beyond these findings, it should be deliberated that humans are trained to make clinical decisions after having assessed the wound comprehensively, going further than mere image inspection. Typically, contextual information is taken into account or as Johansson et al. stated, “Humans think outside the pixels.”^48^ Factors such as the patient’s general state of health, previous medical history, and the patient’s impression on the clinicians all play an important role in the diagnosis.^49–51^ Furthermore, the intuitive cognitive system or deliberative reasoning can determine clinical judgments—particularly in routine cases.^52^

A review article by Hajjaj et al. revealed a long list of non-clinical influencing factors on both patient- and physician-related medical decision making. Important physician-related influencing factors were personal characteristics, time constraints, and the physician’s professional interactions.^53^

Although AI models can be trained on more than just images, including comorbidities and the patients’ medical history, it is difficult to incorporate intuitive information. Therefore, it is widely discussed to employ AI in medicine primarily as a supportive tool for healthcare professionals, not a replacement.^3^^,^^54–57^ However, in medical areas that strongly focus on visual information like tele-dermatology, AI might undertake the first steps of triaging cases.^40^ In other medical areas that require interdisciplinary and interprofessional cooperation, such as in wound care, it is more than desirable to reach a common ground defined by the same diagnosis. Given the heterogeneity of the clinical judgments among the health professionals in this study, AI may contribute to increasing their agreement, which is an assumption supported by the literature.^58^

### Limitations

Maceration is an example of a clinical condition that is not easy to verify by means of an objective test. Therefore, the ground truth determined by a panel of experts may still contain some degree of subjectivity. The same holds true for the labeling of the wound images that were used for training the AI model.

The fact that all of the wound images were sourced from a single wound care center might have had an unintended impact on the outcomes, potentially affecting the variability of the dataset.

On the other hand, specialized university wound centers—from where the data originate—see a great diversity of cases from a wide geographical range.

Due to the same provenance of the images, a potential limitation of this study is that the model’s performance may be overestimated due to the similarities in imaging protocols and backgrounds, which could limit its generalizability to other settings with different workflows or standards. Furthermore, we only considered dermatology residents or specialists and nurses with specialized wound care training as having pertinent formal qualifications. This may represent a bias, as it is conceivable that board-certified surgeons could also possess relevant qualifications in this area, although this is likely to apply to only a small number of individuals.

Our approach deliberately did not account for time pressure and other situational factors of clinical environments that can influence diagnostic accuracy. Therefore, future research should consider evaluating diagnostic performance in settings that more closely replicate the real-world nature of clinical decision making to ensure validity. Detecting maceration in wound images is generally considered relatively straightforward. On top of this, the 30 images selected represented clear cases of maceration and no maceration, making it a rather simple task for both humans and the AI model. It can be hypothesized that the differences in accuracy and reliability among the health professionals become more pronounced as the task and the maceration cases become more difficult. In addition, the response times were not measured, which might have provided further insight into the decision-making process.

### Outlook

The potential of AI-assisted tools to enhance assessment consistency in clinical practice is worth further investigation. When developing AI models and labeling data, it is essential to acknowledge the inherent variability in human judgment, even among experts. Although this study contributed to clarifying the role of human expertise when comparing AI with human decisions, there remains the need to further investigate factors beyond expertise explaining the variance in diagnostic accuracy.

## Conclusion

Our results indicate that while clinicians generally demonstrate a rather high degree of accuracy in diagnosing wound maceration, individual factors significantly influence their performance. In particular, *pertinent formal qualifications* and *self-confidence* in their abilities impact the clinician’s accuracy. It is noteworthy that these 2 characteristics are only moderately correlated, pointing at two more or less distinct constructs.

Insights of this study may also have practical implications. Considering AI systems as sparring partners for clinicians, poor performing clinicians in this seemingly straightforward task might be good candidates for improving their skills with AI.

## Supplementary Material

ocaf116_Supplementary_Data

## Data Availability

The anonymized data that support the findings of this study are available from the corresponding author upon reasonable request.
