# Supplementary material for: Diagnostic accuracy differences in detecting wound maceration between humans and artificial intelligence: the role of human expertise revisited
Source: J Am Med Inform Assoc. 2025 Jul 16;32(9):1425–33. doi: 10.1093/jamia/ocaf116 (PMC12361858; doi:10.1093/jamia/ocaf116)
Supplement: ocaf116_Supplementary_Data [file ocaf116_supplementary_data.docx]

**Appendix A**

**Table A1.** English translated questionnaire.

| **Code** | **Question** | **Answer – Options and Codes** |
| --- | --- | --- |
| **W1** | Can maceration be identified in the wound photograph shown here? | 1 "Maceration present"  0 "No maceration present" |
| **W2** | Can maceration be identified in the wound photograph shown here? |  |
| **W3** | Can maceration be identified in the wound photograph shown here? |  |
| **W4** | Can maceration be identified in the wound photograph shown here? |  |
| **W5** | Can maceration be identified in the wound photograph shown here? |  |
| **W6** | Can maceration be identified in the wound photograph shown here? |  |
| **W7** | Can maceration be identified in the wound photograph shown here? |  |
| **W8** | Can maceration be identified in the wound photograph shown here? |  |
| **W9** | Can maceration be identified in the wound photograph shown here? |  |
| **W10** | Can maceration be identified in the wound photograph shown here? |  |
| **W11** | Can maceration be identified in the wound photograph shown here? |  |
| **W12** | Can maceration be identified in the wound photograph shown here? |  |
| **W13** | Can maceration be identified in the wound photograph shown here? |  |
| **W14** | Can maceration be identified in the wound photograph shown here? |  |
| **W15** | Can maceration be identified in the wound photograph shown here? |  |
| **W16** | Can maceration be identified in the wound photograph shown here? |  |
| **W17** | Can maceration be identified in the wound photograph shown here? |  |
| **W18** | Can maceration be identified in the wound photograph shown here? |  |
| **W19** | Can maceration be identified in the wound photograph shown here? |  |
| **W20** | Can maceration be identified in the wound photograph shown here? |  |
| **P1** | How old are you? | Metric indication (years) |
| **P2** | What gender do you identify with? | 1 "Male"  2 "Female"  3 "Diverse" |
| **P3** | What is your professional qualification? | 1 "Board-certified dermatologist"  2 "Board-certified physician with a different specialization"  3 "Registered nurse / health care professional"  4 "Registered nurse with specialization in wound care (e.g., ICW certification)"  5 "Resident physician in dermatology"  6 "Resident physician in another field"  7 "Medical student"  8 "Allied health professionals"  9 "Other" |
| **P4** | In which sector do you primarily work? | 1 "Inpatient sector (e.g., hospital, rehabilitation clinic)"  2 "Outpatient medical care"  3 "Outpatient nursing care"  4 "Inpatient nursing care (e.g., long-term care, short-term care)" |
| **P5** | How many years of practical professional experience do you have in total? | Metric indication (years) |
| **P6** | Have you ever worked in a role primarily focused on the care and treatment of chronic wounds? | 0 "No"  1 "Yes" |
| **P7** | How confident are you in identifying macerations? | 1 – 10 Likert scale  (1 „not confident at all“ to 10 „very confident“) |

**Appendix B**

**Table B1.** Additional metrics of human and AI diagnostic decisions. Legend: PPV: positive predictive value, NPV: negative predictive value. All 95% confidence intervals were computed according to the Wilson equation.^35^

| **Group** | | **N** | **PPV** | | **NPV** | |
| --- | --- | --- | --- | --- | --- | --- |
|  |  |  | **Result** | **95% CI** | **Result** | **95% CI** |
| **CNN** |  |  | 0.875 | 0.62–0.98 | 0.929 | 0.66–1.00 |
| **Overall human experts** | | 481 | 0.849 | 0.84–0.86 | 0.738 | 0.73–0.75 |
| **Work experience** | |  | | | | |
|  | Short (≤ 5 years) | 68 | 0.814 | 0.79–0.84 | 0.648 | 0.62–0.68 |
|  | Long (> 5 years) | 413 | 0.854 | 0.85–0.86 | 0.753 | 0.74–0.76 |
| **Pertinent formal qualification** | |  | | | | |
|  | No | 232 | 0.800 | 0.79–0.81 | 0.665 | 0.65–0.68 |
|  | Yes | 249 | 0.893 | 0.88–0.90 | 0.807 | 0.79–0.82 |
| **Focus on wound care** | |  | | | | |
|  | No | 215 | 0.819 | 0.81–0.83 | 0.697 | 0.68–0.71 |
|  | Yes | 266 | 0.872 | 0.86–0.88 | 0.771 | 0.76–0.78 |
| **Diagnostic self-confidence** | |  | | | | |
|  | Low | 264 | 0.815 | 0.80–0.83 | 0.692 | 0.68–0.71 |
|  | High | 217 | 0.889 | 0.88–0.90 | 0.794 | 0.78–0.81 |
| **Age** | |  | | | | |
|  | Young (≤ 40 years) | 244 | 0.841 | 0.83–0.85 | 0.726 | 0.71–0.74 |
|  | Old (> 40 years) | 237 | 0.857 | 0.84–0.87 | 0.751 | 0.74–0.76 |
| **Gender** | |  | | | | |
|  | Male | 100 | 0.869 | 0.85–0.89 | 0.743 | 0.72–0.77 |
|  | Female | 381 | 0.843 | 0.83–0.85 | 0.737 | 0.73–0.75 |
| **Healthcare sector** | |  | | | | |
|  | Outpatient | 45 | 0.880 | 0.85–0.90 | 0.784 | 0.75–0.81 |
|  | Inpatient | 436 | 0.845 | 0.84–0.85 | 0.733 | 0.72–0.74 |

**Appendix C**

The multicollinearity between the independent variables was assessed by the variance inflation factor (VIF < 5.0). Homoscedasticity of the residuals was tested using the Breusch-Pagan test, and the Durbin-Watson test was performed to assess the autocorrelation of the residuals. Cook’s distance was calculated to analyze the potential outliers and influencing variables. All the indicators met the requirements of multiple linear regression analysis. Bivariate data exploration via scatterplots showed no hints for non-linear relationships. All tests for the model requirements of multiple linear regression can be seen in Table C1.

**Table C1.** Additional statistics of the multiple linear regression model, including multicollinearity (VIF), autocorrelation (Durbin-Watson), heteroscedasticity (Breusch-Pagan). Significant values (p<0.05) are presented in bold.

| **Coefficient** | **VIF** |
| --- | --- |
| Work experience | 4.48 |
| Pertinent formal qualification | 1.310 |
| Healthcare sector | 1.040 |
| Gender | 1.050 |
| Age | 4.360 |
| Diagnostic self-confidence | 1.530 |
| Focus on wound care | 1.340 |
| **Durbin-Watson test** | 1.946 |
| **Breusch-Pagan test** | 25.478 |

**Appendix D**

**Table D1.** Correlation (phi coefficient) between binary predictors. Significant Chi^2^ values (p<0.05) are presented in bold.

|  | **Work experience** | **Pertinent formal qualification** | **Healthcare sector** | **Gender** | **Age** | **Diagnostic self-confidence** | **Focus on wound care** |
| --- | --- | --- | --- | --- | --- | --- | --- |
| **Work experience** | 1.000 |  |  |  |  |  |  |
| **Pertinent formal qualification** | **0.193** | 1.000 |  |  |  |  |  |
| **Healthcare sector** | 0.007 | **0.139** | 1.000 |  |  |  |  |
| **Gender** | **0.101** | 0.002 | 0.059 | 1.000 |  |  |  |
| **Age** | **0.352** | **0.111** | 0.040 | 0.075 | 1.000 |  |  |
| **Diagnostic self-confidence (binary)** | **0.164** | **0.399** | **0.096** | 0.071 | **0.109** | 1.000 |  |
| **Focus on wound care** | **0.187** | **0.304** | 0.088 | 0.007 | 0.075 | **0.395** | 1.000 |
